# Herman Berendsen’s NMR Research in Groningen and Some Memories from a Fan

**DOI:** 10.1007/s10930-023-10112-w

**Published:** 2023-04-15

**Authors:** Rob Kaptein

**Affiliations:** Bijvoet Center, Utrecht University, Padualaan 8, Utrecht, 3584 CH Netherlands

**Keywords:** NMR spectroscopy, water-biomolecule interactions

## Abstract

This paper describes the scientific work of Prof. Dr. Herman Berendsen on NMR spectroscopy and includes some personal notes. Since 1975, Berendsen and the author were colleagues in the Physical Chemistry group in Groningen for a period of 12 years.

## Introduction

Herman Berendsen (1934–2019) studied experimental physics at Utrecht University. After his Master’s degree in 1957 and an obligatory military service with the Royal Dutch Marine he spent several years (1959–1961) as a research assistant at the Massachusetts Institute of Technology (MIT) in Cambridge (US). There, he became familiar with the emerging technique of Nuclear Magnetic Resonance (NMR) and completed a major part of his doctoral thesis. In 1961 Herman returned to The Netherlands and started to work in the Physical Chemistry group of Prof. Jan Kommandeur at the University of Groningen. He made a rapid career through the academic ranks: he defended his thesis in 1962 (cum laude), became Lector (Associate Professor) in 1963, and Full Professor in 1967. He stayed in Groningen until his retirement in 1999. At the occasion of his valedictory lecture he received a royal decoration: Knight in the Order of the Dutch Lion (see Figure).

It is usually not possible to characterize a person in one word, but for Herman Berendsen this word would be “water”. This applies not only to his scientific work, both in NMR and in Molecular Dynamics (MD), but also for his major hobby, sailing. In the following I will focus on his research in NMR spectroscopy and provide some personal notes. His outstanding work on MD is discussed elsewhere in this issue.

## Herman and NMR Spectroscopy

Herman’s thesis, entitled “An NMR study of collagen hydration”, was a pioneering study of protein - water interaction, summarized in [1]. Using solid-state NMR he found that at high hydration levels the width of the water resonance was highly dependent on the orientation of the collagen fiber with respect to the magnetic field. He could interpret this in terms of a chainlike water structure tightly connected to the fiber. In contrast, at low hydration this dependence was lost, and the water molecules could reorient freely. The article has been cited 366 times, a huge number for a paper from these days.

**Fig. 1:**
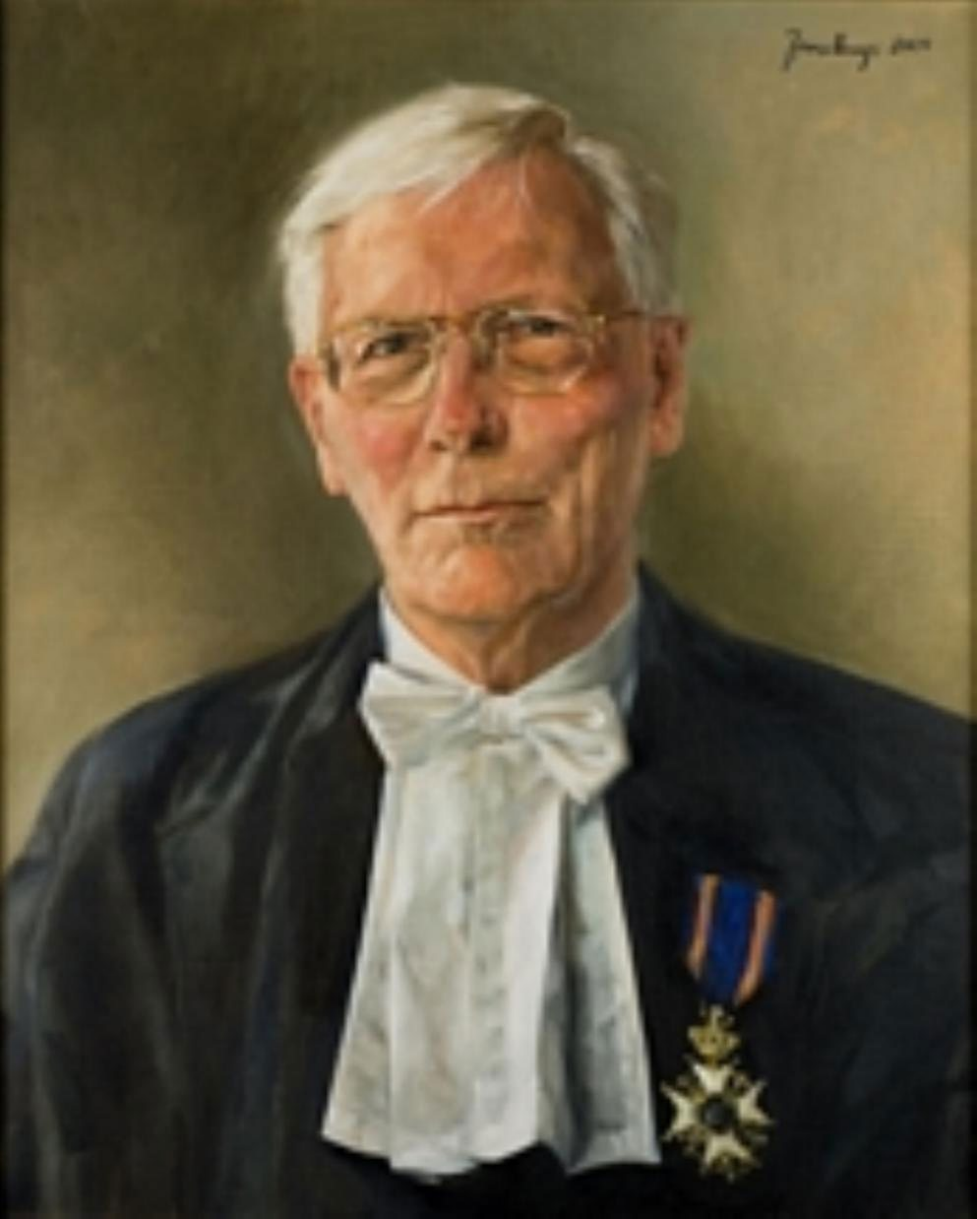
Portrait of Herman Berendsen with the royal decoration that he received in 1999 at the occasion of his retirement.

Other major NMR contributions by Herman include the first correct interpretation of the Na^+^ resonance in biomolecular tissues [2], and his very important discovery of spin diffusion, the dispersion of nuclear spin polarization through cross-relaxation [3]. This was essential for the correct description of proton relaxation processes in NMR spectra of slowly tumbling (bio)molecules in solution.

In the mid-70s Herman’s interests gradually shifted to MD, which would become his major claim to fame. However, surprisingly this turned out to be also useful for the structure determination of proteins based on NMR data [4]. His development of MD simulations with restraints [5] and coupling to an external bath [6] provided the tools for the so-called restrained MD method. In the case of structure determination of proteins by NMR the main restraints are usually proton-proton distances from Nuclear Overhauser Effects (NOE’s), which are easily available from 2D or 3D NOE spectra.

## The Dutch NMR Facility

In the early 70’s it became clear that NMR would become a very important tool for research in chemistry and biology. The Netherlands Organization for Scientific Research (ZWO at the time) made funds available for a Dutch high-field NMR facility. Herman Berendsen applied for such a facility in Groningen and in spite of strong competition his proposal was funded. This allowed the purchase of a 360 MHz NMR spectrometer (the highest field available) and the appointment of support staff. The instrument was delivered late 1974. George Robillard was attracted as a “Lector” (Associate Professor), Klaas Dijkstra as a Technician, and I came early 1975 to Groningen as Supervisor of the facility. Although officially Herman was the PI, he left the management of the facility largely to Klaas and me. This was typical for Herman’s generosity: he could start projects, but after a while leave the further development to his coworkers. Overall, the Physical Chemistry group had a pleasant lack of hierarchy, mainly due to the “Americans”, Kommandeur and Berendsen, but unlike many other research groups in the Netherlands. Young staff members like me were treated as equals and would share in the available money and personnel positions from university sources.

The facility fared well in terms of service to our clients and development of novel NMR methods. However, in NMR one needs a major miracle every 5 or 6 years in the form of funding for the latest equipment. Since even after 10 years that miracle had not happened in Groningen, I decided to accept an offer from Utrecht University to start an NMR group there with a 500 MHz NMR spectrometer and generous funds for staff. That marked the sad passing away of the Groningen facility in 1987.

## Herman the Sailor

After his military service Herman kept a good connection with the yacht-club of the Royal Dutch Marine, where he rented every year in the summer holidays a 12-meter yacht for sailing with his friends and colleagues on the North Sea. The radius of action included Scotland, the south of England with many nice islands, and the various harbors in Normandie and Bretagne. I have joined many of these memorable trips, but sometimes they were pretty tough. For instance, on a 48 h. trip to Scotland we worked on a scheme of 4 h. on/4 h. off, hoping to catch some sleep in the off-time. Interestingly, on the yacht Herman underwent a change of character, from the nice and quiet person he normally was to the strict and rigorous skipper giving orders to his crew. Of course, this was absolutely necessary if one has to order the crew to change sails on bumpy ship at wind-force 7 or 8.

## Conclusions

Herman Berendsen was an outstanding scientist in area’s spanning from mathematics and physics to chemistry and biophysics. The leitmotiv for his research was the interaction of water with biomolecules and how this effects their function. At first, he used mainly NMR spectroscopy combined with other biophysical methods and from the early 70’s he became one of the pioneers of computational methods such as molecular dynamics. Herman was also an excellent and enthusiastic teacher. He was very approachable for his students, postdocs, and colleagues. In short, Herman was the ideal university professor, and he left an unforgettable impression on the people he worked with.
